# From Pig Bite to Endocarditis: An Invasive LA-MRSA Infection

**DOI:** 10.3390/jcdd13100503

**Published:** 2026-10-08

**Authors:** Cindy Madelon Boers, Esther Mintjens, Mirjam Schipper, Tom Brink, Filiz Celik, Thomas O Bergmeijer, Julia M da Silva Voorham

**Affiliations:** 1 Department of Cardiology, Deventer Hospital, 7416 SE Deventer, The Netherlands; 2 Department of Intensive Care, Deventer Hospital, 7416 SE Deventer, The Netherlands; 3 Department of Neurology, Deventer Hospital, 7416 SE Deventer, The Netherlands; 4 Centre for Radiology and Nuclear Medicine, Deventer Hospital, 7416 SE Deventer, The Netherlands; 5 Department of Medical Microbiology, Deventer Hospital, 7416 SE Deventer, The Netherlands

**Keywords:** infective endocarditis, livestock-associated MRSA, ST398, persistent MRSA bacteremia, livestock exposure, *Staphylococcus aureus*

## Abstract

We describe a case of severe invasive infection caused by livestock-associated methicillin-resistant *Staphylococcus aureus* (LA-MRSA, ST398 lineage) in a patient with occupational exposure to pigs. The patient presented with confusion and clinical findings suggestive of bacterial meningitis. Initial cerebrospinal fluid multiplex PCR testing was negative, and empirical antimicrobial therapy was adjusted to intravenous vancomycin to broaden empirical coverage to include staphylococci, including methicillin-resistant strains. Subsequently, both blood and cerebrospinal fluid cultures yielded MRSA. Persistent bacteremia prompted further diagnostic evaluation, revealing native aortic valve infective endocarditis complicated by a perivalvular abscess. Molecular typing confirmed the isolate as LA-MRSA ST398. The patient reported sustaining a pig bite one week before symptom onset, which was considered a plausible portal of entry. Because bacteremia persisted despite treatment with vancomycin and linezolid, antimicrobial therapy was escalated to daptomycin, ceftaroline, and rifampicin, followed by surgical aortic valve replacement. This case highlights the invasive potential of LA-MRSA ST398 and emphasizes the importance of early recognition of persistent bacteremia and metastatic infectious foci. It also underlines that, even in low-endemic countries such as the Netherlands, where MRSA accounts for approximately 2% of all *S. aureus* isolates, LA-MRSA should be considered in patients with relevant livestock exposure and severe invasive disease.

## 1. Introduction

Early recognition of methicillin-resistant *Staphylococcus aureus* (MRSA) is essential in patients presenting with severe invasive infections. This report describes an unusual presentation of invasive LA-MRSA ST398 infection with infective endocarditis, neurological involvement, and multiple septic embolic complications following occupational exposure to pigs. Because invasive MRSA infections are rare in the Netherlands, timely recognition and appropriate treatment may be challenging. The diagnostic work-up, therapeutic management, and clinical decision-making in this complex infection are discussed.

## 2. Case Presentation

A man in his early 40s presented to the emergency department with confusion, right-sided hemianopsia, paresis of the right arm, and fever. Inflammatory markers were markedly elevated (CRP 448 mg/L). Because of the neurological deficits, cranial computed tomography (CT) and lumbar puncture were performed. The CT scan showed a semi-recent cerebral infarction in the left occipital region. Cerebrospinal fluid (CSF) analysis showed pleocytosis (3132 cells/µL) with neutrophilic predominance, consistent with bacterial meningitis.

In accordance with the Dutch national guideline for community-acquired meningitis, treatment with amoxicillin, ceftriaxone, and acyclovir was started in the emergency department. The patient was subsequently transferred to the intensive care unit (ICU). A FilmArray Meningitis/Encephalitis panel (bioMérieux^®^) was negative that same day for the pathogens included in the panel. As *S. aureus* is not included in this panel and remained a relevant differential diagnosis in this severely ill patient, empirical antimicrobial therapy was broadened to include coverage for staphylococci, including methicillin-resistant strains. Amoxicillin was therefore discontinued and replaced with intravenous vancomycin. Within 24 h, both blood and CSF cultures yielded MRSA. Screening cultures of the nasal and rectal mucosa also demonstrated MRSA colonization. Following microbiological confirmation, acyclovir and ceftriaxone were discontinued, vancomycin was continued, and linezolid was added because of its excellent penetration into the central nervous system.

On day 2, the patient was transferred from the ICU to the neurology ward. A collateral history revealed that he worked as a truck driver transporting pigs. Approximately one week before symptom onset, he had been bitten on the hand by a pig. No culture of the bite wound was available. His illness had started with nonspecific symptoms suggestive of a viral infection, including malaise, myalgia, and abdominal pain.

Because of MRSA bacteremia, a work-up for the primary source and metastatic infectious foci was initiated according to the *S. aureus* bacteremia protocol. Transthoracic echocardiography (TTE) showed moderate aortic valve regurgitation without convincing evidence of endocarditis, although image quality was quite poor. MRI of the brain showed ischemic metastatic lesions both supra- and infratentorially, in addition to the previously known cerebral infarction (Figure 1). There were no indications of leptomeningitis, although this could not be excluded.

On day 5 after admission, the patient still had persistent fever and blood cultures remained positive. This suggested a deeper-seated infection. The antibiotic regimen was changed to daptomycin, ceftaroline, and rifampicin. From 48 h after starting the adjusted regimen onward, blood cultures remained negative.

As part of the work-up for metastatic foci, transesophageal echocardiography (TEE) was performed in addition to the earlier TTE. TEE showed a congenitally bicuspid aortic valve with severe regurgitation and extensive vegetations. In addition, PET-CT showed a splenic infarction and multiple soft-tissue lesions, with selected lesions shown in Figure 2.

On day 6, the patient was discussed by the regional multidisciplinary endocarditis team to decide on the indication for and timing of aortic valve replacement. It was weighed that there was a real risk of hemorrhagic transformation of the cerebral lesions during surgery as a result of full heparinization required for cardiopulmonary bypass. Moreover, in the absence of adequate source control, there would be a high risk of re-infection of the new valve prosthesis. On the other hand, without surgery and with inadequate source control, there would be a real risk of new metastatic foci developing and further clinical deterioration. Since day 6 of admission was also the first day with a decline in CRP, it was decided to initially continue antimicrobial therapy and closely monitor the clinical response before scheduling surgery. Day 7 was the first day on which clinical improvement was also observed.

A follow-up TTE was performed later during the admission (day 19) while awaiting aortic valve replacement. This showed that an aortic root abscess had developed (see Figure 3), after which the surgery date was moved forward (day 22). The patient underwent successful surgery with implantation of a Carbomedics Reduced mechanical aortic valve prosthesis. An abscess cavity in the left coronary sinus was debrided and reconstructed using a pericardial patch. Cultures of the excised native valve remained negative, even after prolonged incubation (14 days). At the time of surgery, the patient had received 17 days of daptomycin, ceftaroline, and rifampicin (day 5 to day 22), with blood cultures negative from approximately day 7. Postoperatively, antimicrobial therapy with daptomycin, ceftaroline, and rifampicin was continued for a total of six weeks, calculated from the date of the first negative blood culture. Twenty days after surgery, the patient was discharged home. Final molecular typing of the positive blood cultures identified Livestock-Associated methicillin-Resistant *Staphylococcus aureus* (LA-MRSA, ST398), mecA-positive and Panton-Valentine leukocidin (PVL)-negative.

Five days after completing the antibiotic course, the patient again developed fever and elevated inflammatory markers without other localizing symptoms, while blood cultures remained negative. PET-CT revealed a splenic abscess, for which the same antibiotic regimen was restarted for an additional six weeks (Figure 4). In consultation with the surgical and microbiology teams, it was decided not to perform drainage because of the small size of the abscess (maximum diameter 3 cm) and the high bleeding risk associated with the procedure.

At the time of writing, the patient has been off antibiotics for two months, has returned to work, and continues to recover well.

The chronological sequence of the patient’s clinical course, diagnostic work-up, antimicrobial therapy, and surgical intervention is summarized in Figure 5.

## 3. Discussion

This case underscores the importance of obtaining an early and focused collateral history, with particular attention to occupational and other exposure risks that may point towards uncommon pathogens. According to the Dutch national guideline, empirical treatment of acute native valve endocarditis consists of flucloxacillin. However, in patients with relevant livestock exposure, empirical coverage for MRSA with vancomycin should be considered early.

In the Netherlands, MRSA accounts for approximately 2% of *S. aureus* isolates [1]. However, LA-MRSA carriage is considerably more common among individuals with regular occupational exposure to livestock, with prevalence rates of up to 26.5% reported in livestock-exposed populations [2]. Although LA-MRSA is predominantly associated with asymptomatic colonization in exposed individuals, it is also capable of causing severe invasive infections in humans, including bacteremia, infective endocarditis, and metastatic infectious complications. In contrast, community-acquired methicillin-resistant *Staphylococcus aureus* (CA-MRSA) is more commonly associated with invasive disease and has been linked to greater virulence and enhanced ability to adhere to human cells. Nevertheless, LA-MRSA also possesses cytotoxic properties and, as illustrated by the present case, can cause severe invasive and disseminated infection [3]. The importance of early recognition of *S. aureus* bacteremia is further emphasized by its high mortality, reported to be approximately 10% at one week and 30% at one year [1] with mortality potentially being even higher in MRSA bacteremia.

The patient presented with bacterial meningitis and cerebral ischemia, which were ultimately attributed to septic cerebral emboli secondary to infective endocarditis. Neurological manifestations are a common presenting feature of infective endocarditis. In a Finnish cohort, 47% of patients presented with neurological symptoms, most frequently cerebral emboli (29%) or meningitis (22%), with *S. aureus* being the predominant pathogen [4]. Current meningitis and sepsis guidelines do not routinely recommend early echocardiography to screen for infective endocarditis. Although point-of-care ultrasound (POCUS) may be performed in patients with sepsis, its screening nature limits the detection of endovascular infection [5,6]. Furthermore, the sensitivity of transthoracic echocardiography (TTE) is limited during the early stages of infective endocarditis because vegetations may still be too small to detect [7]. These findings emphasize the importance of maintaining a low threshold for transesophageal echocardiography (TEE) in patients with *S. aureus* bacteremia and neurological manifestations [7,8,9].

Despite a low vancomycin minimum inhibitory concentration (MIC) of 0.5 mg/L, indicating in vitro susceptibility, the patient had persistent fever and blood cultures remained positive after 72 h of treatment with vancomycin and linezolid. Whether this reflected subtherapeutic vancomycin exposure or true treatment failure is relevant when interpreting the clinical course. Therapeutic drug monitoring was performed during continuous vancomycin administration, with steady-state serum concentrations generally ranging from 21 to 22 mg/L, corresponding to an estimated AUC24 of approximately 500–530 mg·h/L and indicating adequate vancomycin exposure. For serious MRSA infections, the recommended AUC24 target is 400–600 mg·h/L when assuming an MIC of 1 mg/L [10]. The persistence of bacteremia despite in vitro susceptibility and adequate vancomycin exposure therefore suggested an uncontrolled high-inoculum endovascular focus rather than inadequate dosing. Persistent bacteremia is a well-recognized feature of invasive MRSA infection, with previous studies reporting a median duration of approximately 7.5 days despite appropriate antimicrobial treatment [11]. However, persistent MRSA bacteremia should not be regarded as an expected finding alone, as it may indicate a deep-seated or uncontrolled endovascular focus and should prompt further diagnostic evaluation and adequate source control.

Linezolid was initially added to vancomycin because of its excellent penetration into the central nervous system, as meningitis was the dominant life-threatening manifestation at presentation and the cardiac source had not yet been identified. We acknowledge that the combination of vancomycin and linezolid has no established role in infective endocarditis and is not supported by current guidelines; moreover, linezolid is bacteriostatic against staphylococci. Daptomycin was not selected initially because of its limited penetration into the cerebrospinal fluid and limited clinical experience in staphylococcal meningitis. Once persistent bacteremia and an endovascular focus became the predominant clinical problem, antimicrobial therapy was escalated to daptomycin, ceftaroline, and rifampicin.

The combination of daptomycin and a β-lactam such as ceftaroline may enhance bactericidal activity through synergistic effects and has been used as salvage therapy for persistent MRSA bacteremia and difficult-to-treat endovascular infections [12]. In this patient, blood cultures cleared within 48 h after initiation of the daptomycin–ceftaroline-based regimen.

The addition of rifampicin on day 5, before valve replacement and in a patient with native valve infection, deserves separate consideration. The rationale included its activity against biofilm-associated and intracellular bacteria, good tissue penetration, and the anticipated implantation of prosthetic material. However, the multicenter, randomized, double-blind ARREST trial found no reduction in relapse, treatment failure, or mortality with adjunctive rifampicin in *S. aureus* bacteremia [13]. Current guidelines recommend rifampicin primarily in prosthetic valve endocarditis and generally only after effective antimicrobial therapy has been established and bacteremia has cleared. Its early introduction in this case of native valve endocarditis therefore represented an individualized, case-specific decision rather than an evidence-based standard approach. This is clinically relevant because rifampicin carries risks of hepatotoxicity, emergence of resistance when bacteremia is not yet controlled, and clinically important drug–drug interactions, including induction of vitamin K antagonist metabolism, which became relevant after implantation of a mechanical valve.

The contribution of rifampicin to the subsequent clinical response cannot be distinguished from that of the daptomycin–ceftaroline combination. Similarly, the negative cultures of the excised valve, even after 14 days of incubation, should be interpreted in the context of the prolonged antimicrobial therapy preceding surgery, including 17 days of daptomycin–ceftaroline therapy as well as earlier vancomycin exposure. Negative valve cultures after prolonged antibiotic treatment do not, however, establish microbiological sterility.

The recurrence of fever and elevated inflammatory markers after completion of therapy, with negative blood cultures and a splenic abscess as the only identified focus, is more consistent with persistence of an incompletely controlled metastatic focus than with microbiological relapse of bacteremia. A splenic infarction was already present on the initial PET-CT (Figure 2) and may subsequently have become infected and evolved into an abscess. Because the duration of antimicrobial therapy was counted from the first negative blood culture, this metastatic focus may have required a longer treatment course, closer radiological follow-up, or source control. This clinical course illustrates that recurrent fever and elevated inflammatory markers after completion of therapy for *S. aureus* endocarditis should prompt repeat imaging for persistent or undrained metastatic foci, even when blood cultures remain negative.

## 4. Conclusions

The pig bite is regarded as the most probable, but not proven, portal of entry, given the patient’s occupational exposure to pigs, the bite on the hand approximately one week before symptom onset, the subsequent nonspecific prodromal illness, and molecular identification of the invasive isolate as LA-MRSA ST398. However, nasal and rectal screening also demonstrated MRSA colonization, which may have preceded the bite and represents a plausible alternative source of the invasive infection, either through translocation from colonized mucosa or an unrecognized skin breach associated with daily animal contact. As no culture of the bite wound was available, the exact portal of entry cannot be established.

This case demonstrates that LA-MRSA ST398, although predominantly associated with livestock colonization, can cause severe invasive disease, including infective endocarditis with widespread metastatic complications. It also highlights the importance of obtaining a detailed occupational and exposure history, particularly in patients with livestock contact. Persistent MRSA bacteremia should prompt a thorough search for an endovascular source and metastatic foci, together with timely source control and individualized antimicrobial therapy.

## 5. Key Points

1. Although MRSA is uncommon in the Netherlands, it should be considered in patients with severe invasive infections, particularly when relevant occupational or livestock exposure is present.

2. A thorough medical and collateral history is essential for identifying potential exposure risks and guiding empirical antimicrobial therapy.

3. In patients with *S. aureus* bacteremia and neurological manifestations, infective endocarditis should be actively excluded, preferably by transesophageal echocardiography when appropriate.

4. Persistent MRSA bacteremia despite appropriate antimicrobial therapy should prompt evaluation for uncontrolled endovascular infection and inadequate source control.

5. Combination therapy with daptomycin and ceftaroline may be considered as salvage treatment for persistent MRSA bacteremia and other difficult-to-treat endovascular infections.

## Figures and Tables

**Figure 1:**
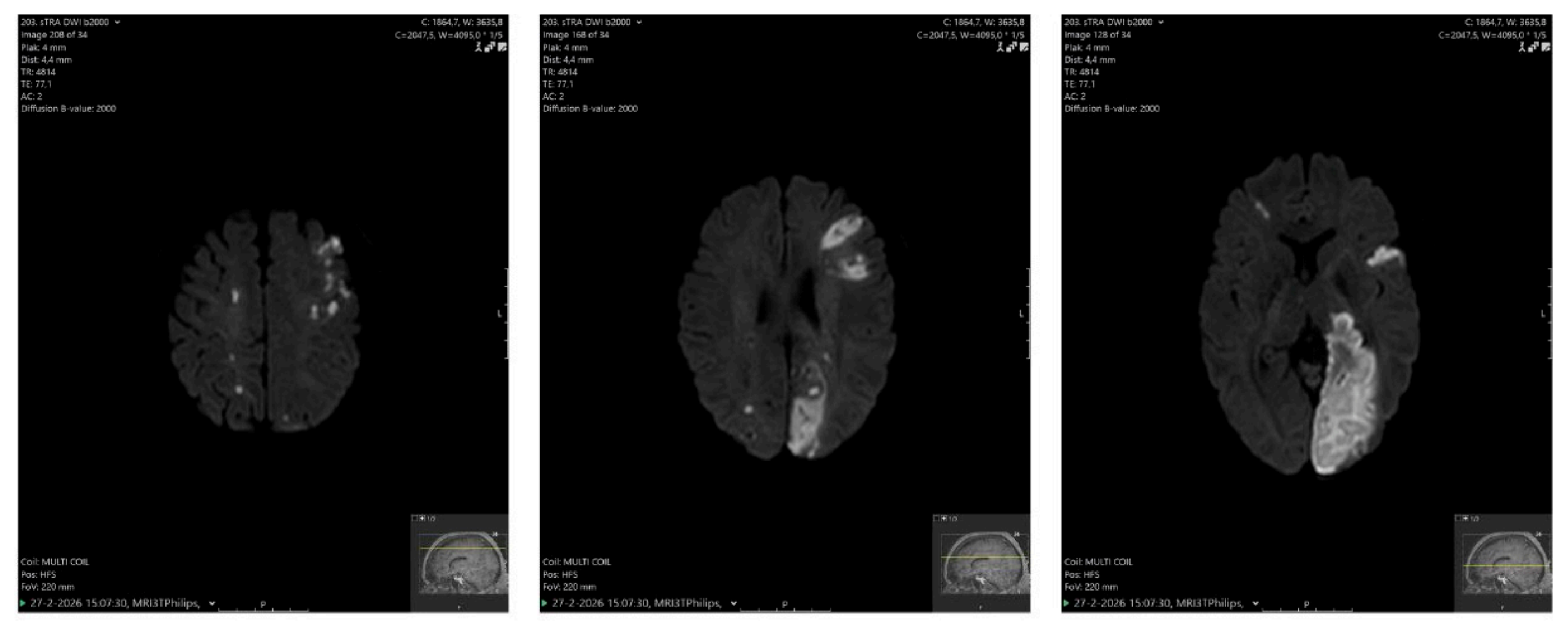
Three axial B2000 image slices of the brain, showing restricted diffusion in multiple vascular territories, including a large area of restricted diffusion in the left occipitotemporal lobe, consistent with septic embolic ischemic foci.

**Figure 2:**
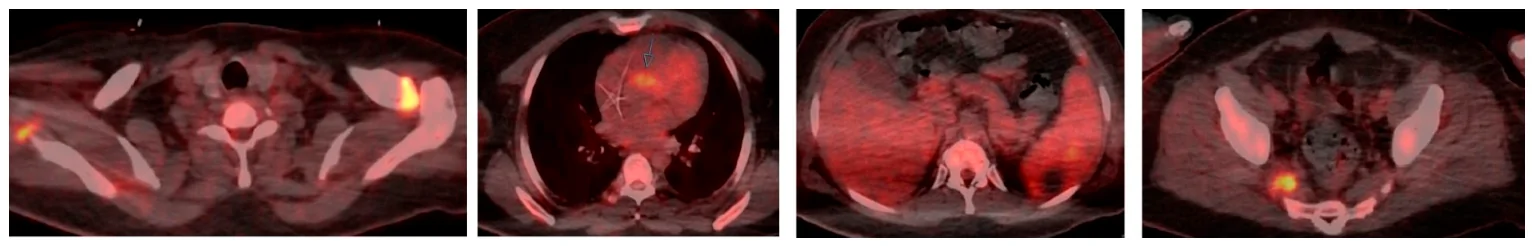
Axial PET-CT images obtained during the initial diagnostic work-up. From left to right: (1) increased focal FDG uptake in the soft tissues around the right scapula and in the left acromioclavicular (AC) joint; (2) moderate FDG uptake at the aortic valve; (3) an FDG-negative area in the spleen, consistent with splenic infarction; and (4) increased focal FDG uptake in the soft tissues of the right pelvis.

**Figure 3:**
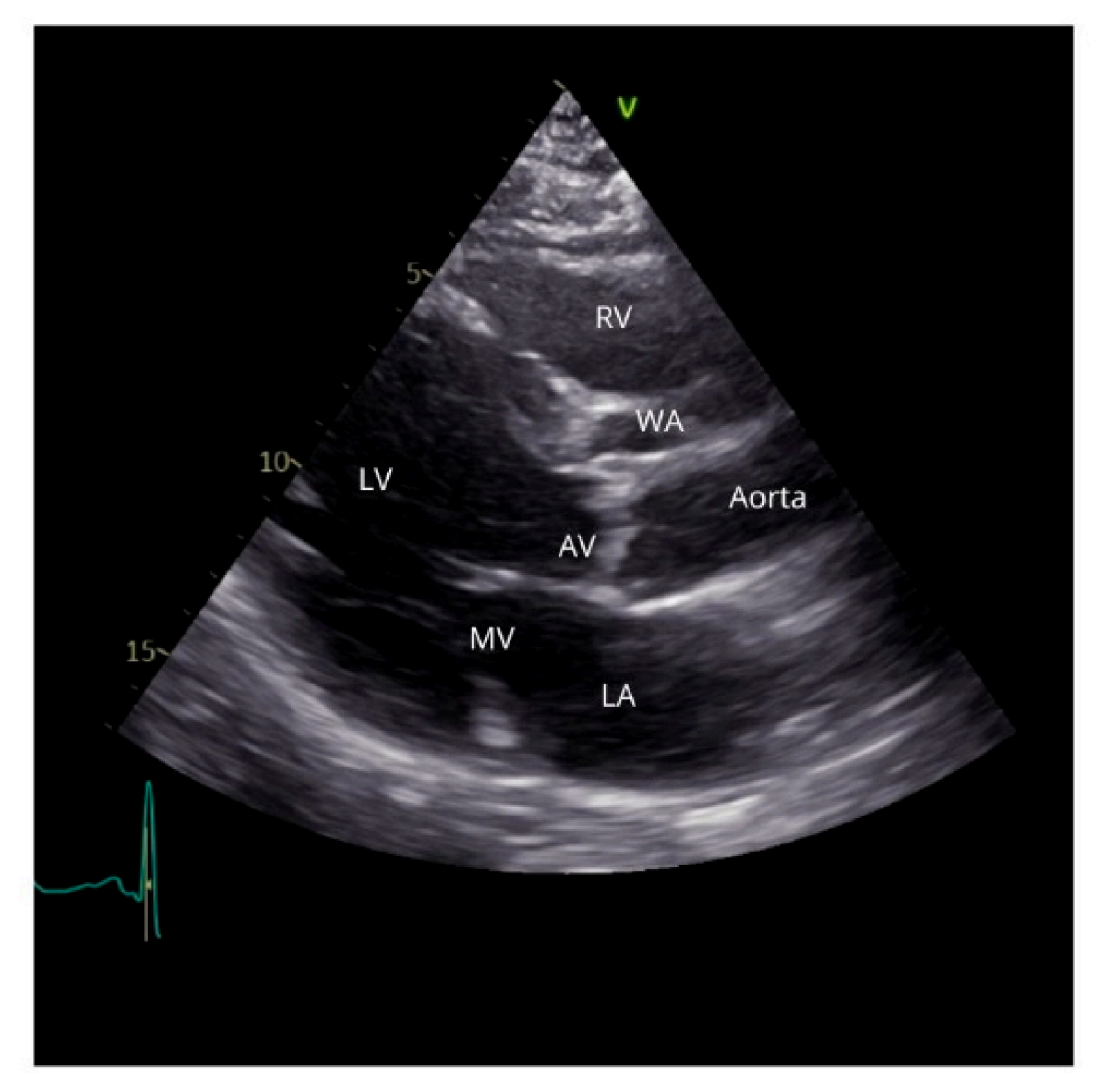
Parasternal long axis of TTE. LA: left atrium; MV: mitral valve; LV: left ventricle; AV: aortic valve; WA: root abscess; RV: right ventricle.

**Figure 4:**
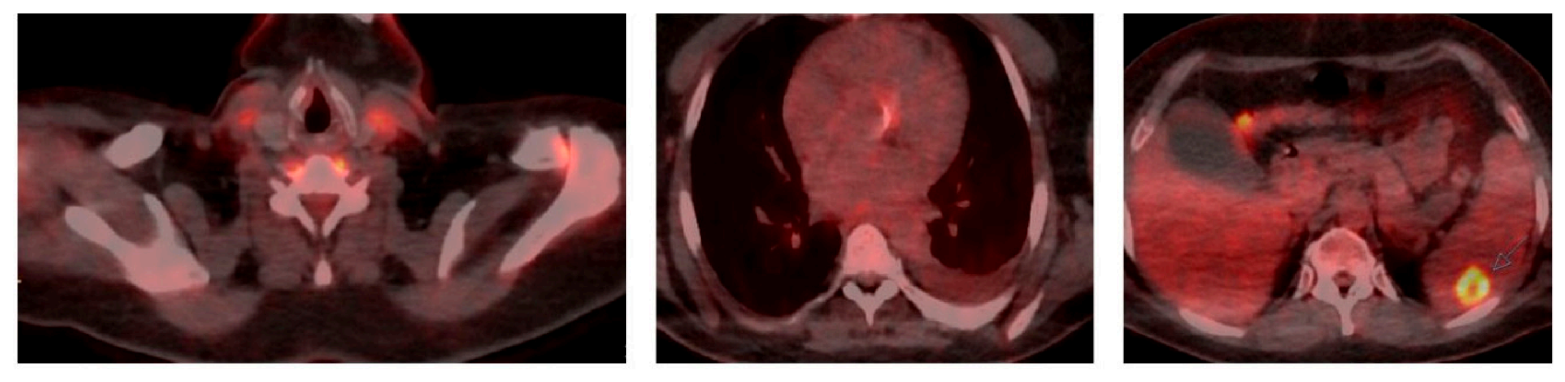
Axial PET-CT images obtained during follow-up. From left to right: (1) no remaining focal FDG uptake in the previously involved soft-tissue sites, with mild residual FDG uptake in the left acromioclavicular (AC) joint; (2) mildly increased FDG uptake at the aortic valve, consistent with postoperative changes; and (3) intense focal FDG uptake in the spleen, suspicious for a splenic abscess.

**Figure 5:**
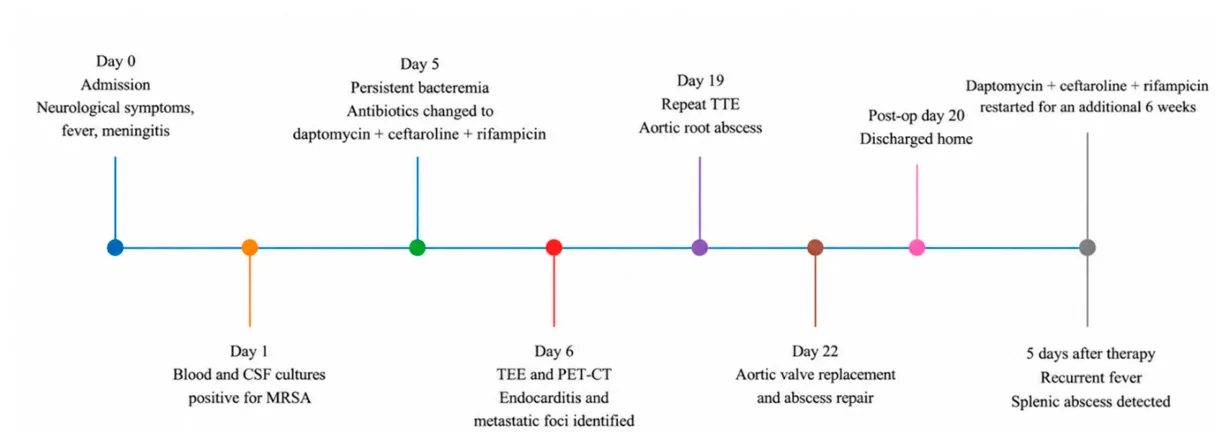
Clinical timeline of invasive LA-MRSA ST398 infection.

## Data Availability

The data presented in this case report are not publicly available due to patient privacy and confidentiality considerations.
